# Carcinoma in situ to invasive breast cancer

**DOI:** 10.18632/oncoscience.170

**Published:** 2015-06-17

**Authors:** Carolien H.M. van Deurzen, John A. Foekens

**Affiliations:** Department of Pathology, Erasmus MC Cancer Institute, Erasmus University Medical Center, Rotterdam, The Netherlands

Invasive breast cancer (IBC) is a heterogeneous disease which can be divided in several molecular subtypes [1] with distinct biological behavior and clinical outcome. Although these subtypes are based on microarray-based gene expression studies, each molecular subtype has an immunohistochemical surrogate: luminal A (ER+ and PR+/Her2−, low Ki-67 index), luminal B (ER+, Her2−, PR− or low and/or high Ki-67 index *or* ER+, Her2+ with any PR expression and Ki-67 index), basal-like (ER−, PR− and Her2−) and Her2-overexpressed (ER−, PR− and Her2+) [2], which has been further refi ned with the addition of CK5/6 and EGFR [3]. The DNA mutation spectrum across these different breast cancer subtypes varies widely [4] and there is increased evidence that genetic alterations, and their prognostic and predictive signifi cance, differ among them.

Ductal carcinoma in situ (DCIS) is regarded as a nonobligate precursor lesion of IBC, sharing a great degree of agreement regarding morphology and genetics [5]. In other words: ER+ and ER− DCIS correlate with respectively ER+ and ER− IBC. Reported incidences of DCIS subtypes suggest that progression of DCIS to IBC differs among subtypes [6]: Triple-negative DCIS seems to have the fastest progression. Luminal A type DCIS seems to progress slower to IBC than luminal B type DCIS. The high incidence of pure Her2+ DCIS compared with Her2+ IBC and the association of Her2+ IBC with an extensive DCIS component might indicate that Her2 amplifi cation does not play a key role in DCIS progression to IBC.

The recent manuscript by Kim et al. [7] focuses on genomic differences between pure DCIS and synchronous DCIS with IBC in order to better understand the mechanisms of DCIS progression. In this interesting study, they compared 6 cases with pure DCIS and 5 pairs of synchronous DCIS and IBC by whole-exome sequencing and copy number profi ling. They concluded that pure DCIS harbored wellknown mutations (e.g. TP53, PIK3CA and AKT1), copy number alterations and chromothripsis, but had significantly fewer driver genes and co-occurrence of mutation/copy number alterations compared with synchronous DCIS with IBC. The authors proposed that although pure DCIS already has some driver mutations, additional changes are needed to progress to IBC. They stated that pure DCIS seems to have a less aggressive genome compared with synchronous DCIS and IBC. However, the study is based on a limited number of cases of different molecular subtypes. All six cases with pure DCIS were ER+, the majority (4 out of 6) was Her2− and all showed low or intermediate Ki-67 index. In conclusion: all pure DCIS cases were of the luminal type, mostly luminal A. On the contrary, the synchronous DCIS-IBC group included a total of fi ve cases, mainly (3 out of 5) ER−. The majority (4 out of 5) showed Her2 amplifi cation and all showed intermediate or high Ki-67 index, which means that this synchronous DCIS-IBC group includes only luminal B and Her2+ cases.

Obviously, the pure DCIS group and the DCIS-IBC group consist of cases with a different molecular subtype; the pure DCIS group mostly luminal A, the DCIS− IBC group mostly luminal B and Her2+. Therefore, the question arises whether the reported genetic differences between these groups are a true refl ection of biological behavior (progression to IBC or not) or that these differences are the result of comparing different DCIS subtypes. In other words, in case one would compare luminal A type pure DCIS to luminal B type or Her2+ pure DCIS, would the results not be similar? In line with this, Kim et al. [7] reported that the ER− and PR− group harbored a higher number of somatic mutations and copy number alterations compared with the ER+ and PR+ group respectively, although this difference was only signifi cant for PR. In addition, the Her2+ cases seem to have more copy number alterations compared with the Her2− cases. Therefore, in our opinion, comparing different molecular DCIS subtypes with different biological behavior (progression or not) does not answer the question whether the reported molecular alterations of the DCIS component are truly responsible for progression since these differences could also be the result of comparing different DCIS subtypes. In order to study true progression-related alterations, we believe it might be preferable to do either paired analyses of synchronous DCIS-IBC or perform analyses of pure DCIS with molecular subtype matched cases with synchronous DCIS-IBC.
